# Kinetic characterization of acetone monooxygenase from *Gordonia* sp. strain TY-5

**DOI:** 10.1186/s13568-018-0709-x

**Published:** 2018-11-03

**Authors:** Osei Boakye Fordwour, George Luka, Mina Hoorfar, Kirsten R. Wolthers

**Affiliations:** 1 Department of Chemistry, University at the British Columbia, Okanagan Campus, 3247 University Way, Kelowna, BC V1V 1V7 Canada; 2 School of Engineering, University at the British Columbia, Okanagan Campus, 3247 University Way, Kelowna, BC V1V 1V7 Canada

**Keywords:** Bayer–Villiger monooxygenase, Acetone monooxygenase, Stopped-flow spectroscopy

## Abstract

Acetone monooxygenase (ACMO) is a unique member of the Baeyer–Villiger monooxygenase (BVMO) family based on its ability to act on small ketones, such as acetone. Herein, we performed a kinetic analysis of ACMO from the propane-utilizing bacterium *Gordonia* sp. strain TY-5 to assess its preference for smaller ketone substrates. Steady state kinetic analysis of ACMO with a range of linear (C3–C7) and cyclic ketones (C4–C6) reveals that the enzyme elicits the highest catalytic efficiency towards butanone and cyclobutanone. Stopped-flow and inhibition studies further revealed that ACMO has a relatively weak binding affinity for the coenzyme with a dissociation constant of 120 μM. We show through mutagenesis that sequence variation in the residue that coordinates to the 2′-phosphate of NADP(H) partially accounts for the weaker binding affinity observed. As for shown for related BVMOs, NADP^+^ stabilizes the C4a-peroxyflavin intermediate in ACMO; however, the rate of its formation is considerably slower in ACMO. The observed rate constant for NADPH-dependent flavin reduction was 60 s^−1^ at 25 °C, and experiments performed with 4(*R*)-[4-^2^H]NADPH confirm that the C4-*pro*-*R*-hydride from the nicotinamide ring is transferred to the FAD. The latter experimental result suggests that the nicotinamide ring rotates within the active site to carry out its two functional roles: reduction of the FAD cofactor and stabilization of the C4a-peroxyflavin adduct.

**Electronic supplementary material:**

The online version of this article (10.1186/s13568-018-0709-x) contains supplementary material, which is available to authorized users.

## Introduction

Baeyer–Villiger monooxygenases (BVMOs) are flavin-containing enzymes that oxidize ketones to esters or lactones, using O_2_ and reducing equivalents from NAD(P)H. They hold promise as versatile biocatalysts given their broad substrate scope and excellent enantio-, and regioselectivity (Leisch et al. 2011; Torres Pazmino et al. 2010). In addition to Baeyer–Villiger oxidations, BVMOs can also oxidize a range of other functional groups including aldehydes, sulfides, amines, phosphines, and selenides and iodide containing molecules (de Gonzalo et al. 2010; Balke et al. 2012; Rehdorf et al. 2009). The majority of BVMOs are type I BVMOs, which bind FAD, elicit a high preference for NADPH, and are composed of a single polypeptide. The substrate scope and catalytic promiscuity varies greatly among the enzyme family. For example, some BVMOs act on bulky steroids, sesquiterpenes or aflatoxins, while the substrate scope of others is limited to smaller (a)cyclic ketones (Kolek et al. 2008).

**Scheme 1:**
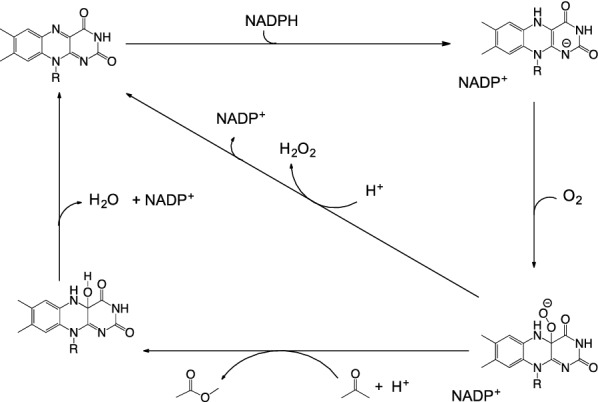
Catalytic mechanism of ACMO

The BVMO catalytic mechanism was formulated from kinetic and spectroscopic analysis of cyclohexanone monooxygenase (CHMO) from *Acinetobacter* sp. NCIMB 9871, one of the more comprehensively studied members of the enzyme family (Scheme 1) (Ryerson et al. 1982; Sheng et al. 2001). Following reduction of oxidized FAD by NADPH, the resulting FADH^−^ reacts with O_2_ to form the C4a-peroxyflavin adduct. In BVMO, the prolonged lifetime of this intermediate is partially attributed to NADP^+^, which remains situated in the active site and is the final product to dissociate from the enzyme. If the enzyme undergoes productive catalysis (i.e. performs a monooxygenation reaction), then the C4a-peroxyflavin adduct attacks the carbonyl group of the ketone substrate, leading to formation of a tetrahedral Criegee intermediate which then collapses to form the lactone and hydroxyflavin intermediate. Release of water from hydroxyflavin returns the FAD cofactor to its oxidized state for another catalytic cycle. In the absence of substrate, the C4a-peroxyflavin adduct can become protonated and collapse to form H_2_O_2_, leading to uncoupled NADPH oxidation.

Acetone monooxygenase (ACMO) is an example of a Type I BVMO that functions in the catabolism of small organic ketones. The enzyme was initially isolated from *Gordonia* sp. strain TY-5, a Gram-positive bacterium capable of aerobic growth with gaseous propane as the sole carbon source (Hausinger 2007). The bacterium encodes an NADH-dependent dinuclear-iron-containing multicomponent monooxygenase that converts propane to 2-propanol and three secondary alcohol dehydrogenases that oxidize 2-propanol to acetone. ACMO, encoded by the *acmA* gene is part of a bicistronic operon that also includes the *acmB* gene. ACMO was shown to convert acetone to methyl acetate, while the gene product of *acmB,* an esterase, hydrolyzes methyl acetate to methanol and acetate (Kotani et al. 2007).

ACMO appears to be unique among BVMOs based on its ability to act on acetone. More well studied BVMO family members such as CHMO and phenylacetone monooxygenase from *Thermobifida fusca* (PAMO) are not able to catalyze the oxidation of this smallest ketone (Fraaije et al. 2005; Donoghue et al. 1976). Our group was interested in examining AMCO’s catalytic efficiency towards acetone to assess its potential to be used as a biosensor, for example in the detection of ketone bodies in the salvia of diabetics. Herein, we measured the enzyme’s catalytic efficiency for acetone, relative to larger linear and cyclic ketones. The stability of the enzyme at various temperatures was also measured along with the pH-dependence of its activity. Stopped-flow analysis of the reductive half reaction with 4(*R*)-[4-^2^H]NADPH supports transfer of the *pro*R-hydrogen, while pre-steady state kinetic analysis of the oxidative half reaction reveals that the C4a-peroxyflavin intermediate is a less stable intermediate in ACMO. Finally molecular modeling revealed structural variation in the coenzyme binding pocket that results in weaker NADP(H) binding affinity.

## Materials and methods

### Materials

NADPH, NADP^+^, ketone substrates, xanthine, xanthine oxidase, methyl viologen, and benzyl viologen were purchased from Sigma. Restriction enzymes were purchased from New England Biolabs, and *PfuTurbo* DNA polymerase was obtained from Agilent Technologies. All other chemicals and media were purchased from VWR. 4(*R*)-[4-^2^H]NADPH was synthesized and purified following a published protocol (Bowman et al. 2010).

### Construction of the ACMO expression vector

The cDNA encoding ACMO was synthesized by GenScript (Piscataway, NJ, USA; GenBank Accession number MH880286).). The coding sequence was optimized for expression in *E. coli* and subcloned into pET23a(+), which resulted in a C-terminal hexahistidine tag. This construct did not result in the overexpression of a soluble form of ACMO, so the opportunity was taken to sub-clone the ACMO cDNA into the pGEX4T1 vector, which places a glutathione *S*-transferase (GST) motif onto the N-terminus of the protein. The forward (5′-CG**G GAT CC**A TGA GCA CGA CGA CGC TGG-3′) and reverse (5′-CCG **CTC GAG** TTA CGA CAG TGC GAA ACC-3′) primers used to amplify the ACMO coding sequence harbored *Bam*HI and *Xho*I restriction enzyme sites (in bold), respectively. The cDNA for ACMO was PCR amplified using *PfuTurbo* DNA polymerase, digested with *Bam*HI and *Xho*I and ligated into pGEX4T1 also cut with the same restriction enzymes. The resulting construct (pGEX-ACMO) was transformed into *E. coli* DH5α for protein expression.

### Construction of ACMO H325K and A443Δ variants

The H325K and A443Δ variants were constructed using the QuikChange II site-directed mutagenesis kit from Agilent Technologies using the pGEX-ACMO vector as a template. The forward and reverse primers used for the H325K substitution are as follows: 5′-GAT TAC GGC TTC GGT ACC AAA CGC GTG CCG CTG GAA AC-3′ and 5′-GAT TAC GGC TTC GGT ACC TTT CGC GTG CCG CTG GAA AC-3′. The oligonucleotides for the deletion of A433 are 5′-CCG CTG GCT CCG AGC GCT CTG TCG AAT ATG-3′ and 5′-CAT ATT GCA CAG AGC GCT CGG AGC CAG CGG-3′. The successful incorporation of lysine at position 325, the deletion of A433, and the absence of any unintended sequence alterations was confirmed by DNA sequencing at the NAPS DNA Sequencing Laboratory at University of British Columbia, Vancouver, Canada.

### Expression and purification of ACMO and the H325K and A443Δ variants

An overnight culture of DH5α harbouring the pGEXACMO plasmid was used to inoculate six 1.5 L flasks of Terrific Broth supplemented with ampicillin (100 μg/mL). Cultures were grown at 30 °C with agitation, set at 200 rpm, to an OD_600_ of 0.8. Recombinant protein expression was induced with the addition of 0.2 mM of IPTG to the cell cultures. Following continued growth at 25 °C for 16 h, the cells were harvested by centrifugation (6000×*g* for 15 min). The cell pellet was frozen at − 80 °C until purification.

All purification steps were performed on ice or at 4 °C. The frozen cell pellet (~ 25 g) was resuspended in 200 mL of GST bind/wash buffer (10 mM Na_2_HPO_4_, 1.8 mM KH_2_PO_4_, 0.14 M NaCl, 2.7 mM KCl, 1 mM EDTA, 1 mM DTT, pH 7.5) containing 10 μg/mL of benzamidine and 1 mM phenylmethylsulfonyl fluoride. The cells were lysed and the genomic DNA was sheared by sonication and then the cell suspension was centrifuged (38,000×*g* for 60 min). The clarified cell extract was applied to a 5.5 × 4.0 cm column containing glutathione Sepharose 4B (GE Healthcare Life Sciences) equilibrated with GST bind/wash buffer. The column was washed with 1 L (~ 10 column volumes) of GST bind/wash buffer. The GSTACMO chimera was eluted with 50 mM Tris–HCl, pH 8.0 supplemented with 1 mM dithiothreitol and 10 mM glutathione. The eluate was dialyzed against 4 L of GST bind/wash buffer containing 1 mM EDTA and 1 mM dithiothreitol for 16 h with thrombin to cleave the GST tag. To separate ACMO from the uncut GSTACMO chimera and thrombin, the dialyzed protein was applied to a glutathione Sepharose 4B (2.5 × 3.0 cm) column equilibrated with GST bind/wash buffer. The column was then washed with 50 mL of the GST bind/wash buffer with 10% glycerol (v/v). The yellow fractions were collected and the protein was concentrated using a centrifugal concentrator with a 30 kDa cutoff filter. ACMO was then applied to a Q-Sepharose column (2.6 × 14 cm) equilibrated with buffer A (50 mM Tris–HCl pH 7.5, 10% glycerol). The protein was eluted with a linear gradient to 50 mM Tris–HCl pH 7.5, 10% glycerol, 0.5 M NaCl. The eluted protein was concentrated and stored at − 80 °C in 20% glycerol (v/v).

### Determining the molar absorption coefficient of ACMO

The UV–visible absorbance spectrum of ACMO was recorded between 700 and 200 nm in 50 mM HEPES pH 7.5 at 25 °C. The flavoprotein (1.2 mL) was then added to 0.3 mL of a 10% solution of SDS to obtain a final concentration of 0.2% SDS. Changes in the spectra of the flavin cofactor were recorded at 4 min intervals until a constant absorbance spectrum was obtained. The protein solution was then centrifuged at 14,000 rpm for 10 min in a microcentrifuge and the spectrum of the FAD liberated from the protein was measured. The concentration of free FAD released from the protein was calculated using a molar absorptivity of free FAD (Ɛ_450_ = 11,300 M^−1^ cm^−1^). The molar absorption coefficient of ACMO was calculated to be Ɛ_443_ = 12,200 M^−1^ cm^−1^ after correcting for the dilution of the protein.

### NADP^+^ binding constant determination

UV–visible absorbance spectroscopy was used to determine the equilibrium dissociation constant for NADP^+^. The assays were performed on a Perkin Elmer Lambda 25 UV–visible spectrophotometer in 50 mM HEPES pH 7.5 at 25 °C. NADP^+^ was sequentially added to an absorbance cuvette containing 50 µM of flavoprotein, achieving NADP^+^ concentrations ranging from 0 to 200 μM. The change in absorbance at a given wavelength was fit to the quadratic binding isotherm (Eq. 1).

1 $$\Delta A = \left( {\frac{{\Delta A_{max} }}{{2E_{o} }}} \right)\left\{ {E_{o} + L_{o} + K_{d} - \left[ {\left( {E_{o} + L_{O} + K_{d} } \right)^{2} - 4E_{o} L_{o} } \right]^{{{\raise0.7ex\hbox{$1$} \!\mathord{\left/ {\vphantom {1 2}}\right.\kern-0pt} \!\lower0.7ex\hbox{$2$}}}} } \right\}$$

where L_*o*_ is total NADP^+^ concentration; *E*_*o*_ is total enzyme concentration; Δ*A* is the change in absorbance, Δ*A*_max_ is the maximum change in absorbance; and *K*_d_ is the dissociation constant for the GSTACMO-NADP^+^ complex.

### Determining the reduction potential of GSTACMO

The reduction potential of GSTACMO was determined by Massey’s method (Massey 1991). The method relies on the near simultaneous reduction of GSTACMO and a reference dye with a known reduction potential by reducing equivalents derived from xanthine oxidase and xanthine. The reference dye used in the GSTACMO reduction was indigo disulfonate, which has a reduction potential of *E*_m_^0^ = − 116 mV at pH 7.0. The titration of GSTACMO was performed at 25 °C in a total volume of 1 mL in an anaerobically maintained glove box in 50 mM HEPES, pH 7.0, that was made anaerobic by the same method described for the stopped-flow experiments. A concentrated 2 mL stock of GSTACMO (36 μM) was introduced into the glove box and gel filtered over a 10 mL PD10 column (Bio-Rad) equilibrated with anaerobic buffer (50 mM HEPES, pH 7.0). Stock solutions of benzyl viologen, indigo disulfonate, xanthine and xanthine oxidase were prepared by dissolving lyophilized powders of each in anaerobic buffer in the glove box. The reaction contained 20 µM GSTACMO, 20 µM indigo disulfonate, 0.5 mM xanthine, and 5 µM benzyl viologen. Following the addition of 0.25 µM xanthine oxidase, the reaction mixture was placed in a Lambda 265 spectrophotometer (Perkin Elmer) also housed in the glove box, and reduction of the enzyme and dye was monitored over 2 h by recording the absorption spectrum of the reaction mixture every minute. The concentrations of the oxidized and reduced enzyme (*E*_ox_ and *E*_red_) throughout the titration were determined at 458 nm (the isosbestic point of indigo disulfonate), while the concentration of the dye (both the oxidized and reduced forms; *D*_ox_ and *D*_red_) was determined at 610 nm, where oxidized and hydroquinone forms of the FAD cofactor do not absorb. The reduction potential of the enzyme (*E*_e_) and the dye (*D*_d_) were calculated from the following equations:

2 $$E_{e} = E_{e}^{^\circ } - \frac{0.0592}{{n_{e} }}\log \left( {\frac{{E_{red} }}{{E_{ox} }}} \right)$$

3 $$E_{d} = E_{d}^{^\circ } - \frac{0.0592}{{n_{d} }}\log \left( {\frac{{D_{red} }}{{D_{ox} }}} \right)$$

where *n* is the number of electrons. At equilibrium, *E*_d_ is equal to *E*_e_ and Eqs. 1 and 2 can be rearranged to the following equation:

4 $$\log \left( {\frac{{E_{red} }}{{E_{ox} }}} \right) _{ = } \frac{{n_{e} \left( {E_{e}^{^\circ } - E_{d}^{^\circ } } \right)}}{0.0592} + \left( {\frac{{n_{e} }}{{n_{d} }}} \right){ \log }\left( {\frac{{D_{red} }}{{D_{ox} }}} \right)$$

Equation 3 enables the reduction potential of GSTACMO, $E_{e}^{^\circ }$ to be determined from the y-intercept of a plot of log(*E*_red_/*E*_ox_) versus log(*D*_red_/D_ox_).

### Steady-state kinetic assays

The steady-state parameters of ACMO and GSTACMO were determined for a variety of linear and cyclic ketones by following the rate of oxidation of NADPH at 340 nm (ΔƐ_340_ = 6.22 mM^−1^ cm^−1^). The reactions were performed in a total volume of 1 mL in air-saturated 50 mM HEPES buffer, pH 7.5 at 25 °C using a Lambda 25 UV–visible spectrophotometer (Perkin Elmer) placed on the laboratory bench. The steady-state kinetic parameters were determined by measuring the initial velocity for the oxidation of various ketones in 1 mL reaction mixtures that contained 100 µM NADPH and variable concentrations (0.1–5 mM) of the ketone substrate. The Michaelis constant for NADPH was determined with variable concentrations of NADPH and a fixed concentration of butanone (200 μM). All steady-state reactions were initiated with 23–45 nM of ACMO or GSTACMO. The initial velocities were plotted as a function of substrate concentration and fitted to the Michealis Menten equation using nonlinear least squares analysis with the computer program Origin 8.0 (OriginLab). The NADP^+^ inhibition assays were performed with variable NADP^+^ (0–1 mM) and NADPH (0.2–100 μM) concentrations in the presence of 200 μM butanone. The inhibition data were fitted to the competitive inhibition equation using Origin 8.0. The rate of uncoupled NADPH oxidation was determined by measuring absorbance changes at 340 nm in the presence of 100 μM NADPH and in the absence of a ketone substrate.

### Stability assays and pH-dependence

The thermostability of GSTACMO was analyzed by incubating the purified enzyme (0.5 μM) at a set temperature (ranging from 5 to 45 °C) for 1 h in 50 mM HEPES, pH 7.5. After incubation the samples were placed on ice and then measured for activity by following the rate of NADPH oxidation spectrophotometrically at 340 nm. Residual activity was measured at 25 °C in 50 mM HEPES, pH 7.5 containing 100 μM NADPH and 200 μM butanone. The reactions were initiated with the addition of 25 nM GSTACMO. The activity of GSTACMO was measured at a range of pH values (5.5–9.0) using a three-component buffer containing 50 mM each of MES, HEPES and CHES. Each 1 mL contained 100 μM NADPH, 200 μM butanone and 50 nM of GSTACMO. The bell-shaped pH profiles where catalysis requires the ionization of a group with a low p*K*_a_ and the protonation of a group having a higher p*K*_a_ were fit to the equation:

5 $$\log Y = \log \left[ {\frac{{Y_{H} }}{{1 + H/K_{a2} + K_{a1} /H}}} \right]$$

where *Y* is the observed velocity, *Y*_H_ is the velocity when both ionizable groups are in their preferred ionization state for maximal activity, and *K*_a1_ and *K*_a2_ are the dissociation constants for the groups that ionize at low and high pH, respectively. The activity at each pH and temperature was measured in triplicate.

### Pre-steady state kinetics

Pre-steady state kinetic assays were performed under anaerobic conditions using a SF-61DX2 stopped-flow apparatus (TgK Scientific). The sample-handling unit of the stopped-flow is housed in a glove box (Belle Technology) with O_2_ concentration < 5 ppm. The reductive and oxidative half reactions were performed at 25 °C in 50 mM HEPES, pH 7.5 with 20% glycerol. The buffer was made anaerobic by purging with nitrogen gas for 2 h followed by a 16 h equilibration in the glove box. Stock solutions of NADPH and NADP^+^ were prepared by dissolving lyophilized powders of the coenzyme in anaerobic buffer. The enzyme was diluted to the appropriate concentration with anaerobic buffer. The reductive-half reaction (following NADPH-dependent reduction of oxidized GSTACMO) was monitored by rapidly mixing the oxidized enzyme with an equal volume of NADPH at a concentration that was at a minimum sevenfold higher than the protein concentration so as to maintain pseudo-first order conditions. Changes in the flavin absorbance spectra were monitored at 443 nm using a photomultiplier and the absorbance traces were fitted to a single exponential equation using Kinetic Studio (TgK Scientific). The concentration of the protein and cofactors were each diluted twofold in the observation cell. The NADPH concentration dependence profiles were fit to the following hyperbolic equation:

6 $$k_{obs} = \frac{{k_{red} \left[ {NADPH} \right]}}{{K_{d} + \left[ {NADPH} \right]}}$$

where *k*_red_ is the limiting rate constant of flavin reduction and *K*_d_ is the dissociation constant for NADPH.

The oxidative half-reaction was monitored under NADPH uncoupling conditions (in the absence of ketone substrate) and under normal turnover conditions (in the presence of butanone). In the first experiment, an anaerobic solution of GSTACMO (20 μM) was reduced with the addition of an equimolar amount of NADPH. NADP^+^ (at a final concentration of 500 μM) was added to the pre-reduced enzyme and then this solution was rapidly mixed with an equal volume of air-saturated 50 mM HEPES pH 7.5, 20% glycerol. The second experiment was performed under the same conditions except that 400 μM butanone was added to the aerated buffer. Butanone was chosen as a substrate as it elicited the highest catalytic efficiency for ACMO. Changes in the flavin spectra were monitored at multiple wavelengths using a photodiode array detector or at individual wavelengths with a photomultipier. Typically, five absorbance traces in single wavelength mode were fitted to a standard single or double exponential equation to extract the observed rate constants.

## Results

### Purification and spectral characterization of ACMO

**Fig. 1:**
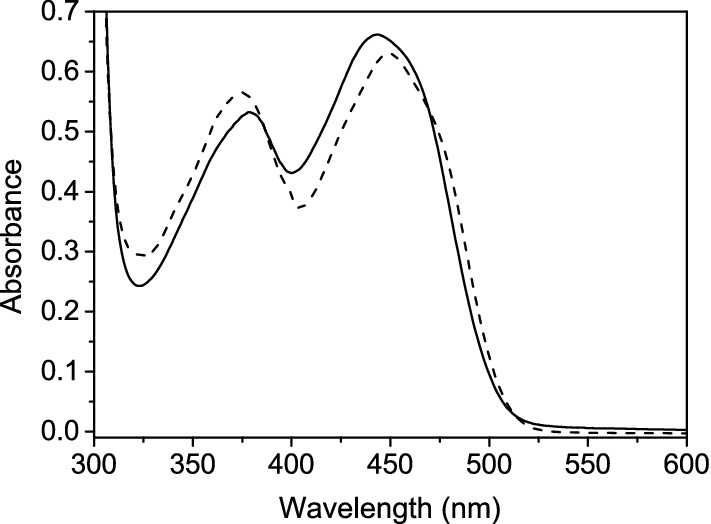
UV–visible absorbance spectra of ACMO showing the diagnostic flavin peaks at 380 and 440 nm. The black line denotes the spectrum of ACMO while the dashed line is the released FAD cofactor following incubation of ACMO in 0.2% SDS for 10 min at 25 °C in 50 mM HEPES–NaOH pH 7.5

Recombinant ACMO was expressed as a GST fusion protein, which enabled the first purification step to involve glutathione affinity chromatography. The GST-tag appeared to stabilize the flavoprotein as removal of the tag following thrombin cleavage caused the protein to slowly precipitate in 50 mM Tris–HCl, pH 7.5 at 4 °C. To prevent precipitation, glycerol (10% v/v) was added to the flavoenzyme prior to loading on to the Q-Sepharose column. Following elution from the Q-Sepharose column, the protein was shown to have a molecular mass of ~ 60 kDa (close to the calculated molecular mass of 59,780 Da) and was 90% homogeneous, as deduced from a Coomassie Blue stained SDS-PAGE gel (Additional file 1: Fig. S1). Approximately, 10 mg of protein was obtained from 1 L of bacterial culture. The UV–visible absorbance spectrum of ACMO showed absorbance maxima at 380 and 443 nm, typical of flavins and flavoproteins (Fig. 1). Release of the FAD cofactor from ACMO in the presence of 0.2% SDS, enabled the extinction coefficient of ACMO (ε_443 nm_ = 12,200 ± 490 M^−1^ cm^−1^) to be calculated from the known extinction coefficient of free FAD.

### Steady state assays

The steady state kinetic parameters of ACMO were determined in 50 mM HEPES, pH 7.5 at 25 °C for a number of linear and cyclic ketones (Table 1). The rate of NADPH oxidation (measured by the absorbance change at 340 nm) in the presence of various ketones was used to access the enzyme’s substrate scope. It has been reported for PAMO and CHMO that either release of NADP^+^ or dehydration of the C4a-hydroxyflavin is the rate-determining step in catalysis, not oxidation of the ketone substrate (Sheng et al. 2001; Torres Pazmino et al. 2008). As a result these enzymes elicit similar turnover numbers with a broad selection of substrates (Donoghue et al. 1976; Mascotti et al. 2013, 2014). We observe a similar phenomenon with ACMO, as Table 1 shows that the enzyme exhibits narrow range of *k*_cat_ values (1.4–4.3 s^−1^) for a variety of ketone substrates, indicating that like other BVMOs, oxygen insertion into the substrate is not the rate-determining step of catalysis. As shown in Table 1, ACMO elicits low *K*_m_ values (and correspondingly high *k*_cat_/*K*_m_ values) for small (a)cyclic ketones like butanone and cyclic butanone. The exception is acetone, which had a 500-fold higher *K*_m_ and a 700-fold lower *k*_cat_/*K*_m_ compared to butanone. For the acyclic ketones, lengthening of the chain or branching of the substrate with the addition of methyl groups resulted in an increase in the *K*_m_, suggesting that these bulkier substrates bind more weakly to the enzyme. Likewise, a progressive increase in the size of the ring of the cyclic ketone led to a gradual increase in *K*_m_ and a decrease in *k*_cat_/*K*_m_. ACMO exhibited no activity towards cycloheptanone. However, ACMO did show activity for phenylacetone and bicyclo[3.2.0]hept-2-en-6-one.

**Table 1:** Steady-state kinetic parameters of ACMO and GSTACMO

Enzyme	Variable substrate	*k*_cat_ (s^−1^)	*K*_m_ (µM)	*k*_cat_*/K*_m_ (M^−1^ s^−1^) × 10^3^
ACMO	NADPH	2.0 ± 0.1	6.7 ± 0.8	300 ± 36
ACMO	Acetone	1.4 ± 0.2	170 ± 11	8.5 ± 0.59
ACMO	Butanone	2.1 ± 0.1	0.34 ± 0.03	6000 ± 550
ACMO	2-Pentanone	1.9 ± 0.1	0.37 ± 0.06	4800 ± 760
ACMO	2-Heptanone	3.9 ± 0.1	1.5 ± 0.1	2600 ± 130
ACMO	3-Methylbutanone	2.2 ± 0.1	4.4 ± 0.5	510 ± 55
ACMO	2,4-Dimethyl-3-pentanone	1.5 ± 0.1	1500 ± 220	1.0 ± 0.15
ACMO	Cyclobutanone	2.0 ± 0.1	1.5 ± 0.2	1400 ± 190
ACMO	Cyclopentanone	4.3 ± 0.1	120 ± 11	35 ± 3.2
ACMO	Cyclohexanone	3.6 ± 0.2	2400 ± 400	1.5 ± 0.3
ACMO	Bicyclo[3.2.0]hept-2-en-6-one	1.5 ± 0.1	6.7 ± 1.3	220 ± 45
ACMO	Phenylacetone	1.0 ± 0.1	8.9 ± 1.5	112 ± 20
GSTACMO	Cyclohexanone	2.8 ± 0.1	2300 ± 380	1.2 ± 0.2
H325K	NADPH	1.6 ± 0.1	0.48 ± 0.05	3250 ± 320
H325K	Butanone	1.6 ± 0.1	0.48 ± 0.05	3250 ± 320

The experiments were performed in 50 mM HEPES–NaOH pH 7.5 at 25 °C as described in “Materials and methods”. Butanone was present at a fixed saturating concentration of 200 μM in steady-state experiments where NADPH served as the variable substrate. For experiments where the ketone served as the variable substrate, NADPH was present at 100 µM

The rate of uncoupled NADPH oxidation was relatively high at 0.26 s^−1^, ~ tenfold lower than that observed in the presence of saturating amounts of the ketone substrate (Table 2). By comparison, the rate of uncoupled NADPH oxidation in PAMO is 0.02 s^−1^ (Torres Pazmino et al. 2008). The relatively high rate of NADPH oxidase activity for ACMO suggests that the C4a-peroxyflavin is less stable in this enzyme and more prone to decay to H_2_O_2_. The Michaelis constant (*K*_m_) for NADPH, determined in the presence of 200 μM butanone was 6.7 ± 0.8 μM. ACMO is highly specific for NADPH as no activity was detected with NADH as the reductant. NADP^+^ was found to be a poor competitive inhibitor of ACMO with a *K*_i_ of 166 ± 13 μM. By comparison, PAMO and CHMO elicit *K*_i_ values of 2.7 μM and 35 μM, respectively, for the oxidized coenzyme (Ryerson et al. 1982; Torres Pazmino et al. 2008). Finally, the GST tag does not appear to adversely affect the kinetic behavior of the enzyme as GSTACMO (ACMO with a fused N-terminal GST tag) elicited similar *k*_cat_ and *K*_m_ with cyclohexanone compared to ACMO. Given that chimeric protein elicits similar steady state kinetic properties as ACMO and is more stable, subsequent kinetic, thermodynamic and binding studies described below were performed on GSTACMO.

**Table 2:** Additional kinetic parameters for ACMO

Enzyme	Parameters	Measured values
ACMO	*K*_I (NADP+)_	166 ± 13 µM
H325K	*K*_I (NADP+)_	27 ± 6 µM
ACMO	*k*_unc_ (s^−1^)	0.26 ± 0.02 s^−1^
ACMO	*k*_red_ (s^−1^)	59 ± 3 s^−1^
ACMO	*K*_d (NADPH)_	120 ± 14 μM

The inhibition constant for NADP^+^, *K*_i (NADP+)_ was determined in the presence of 200 μM butanone. The turnover rates for uncoupled NADPH oxidation (*k*_unc_) were measured with 100 μM NADPH. The limiting rate constant for NADPH-dependent flavin reduction (*k*_red_) and the dissociation constant for NADPH (*K*_d__(NADPH)_) were determined by fitting Eq. 6 to the data shown in Fig. 4a. All reactions were performed at 25 °C in 50 mM HEPES–NaOH, pH 7.5

### Molecular modelling

**Fig. 2:**
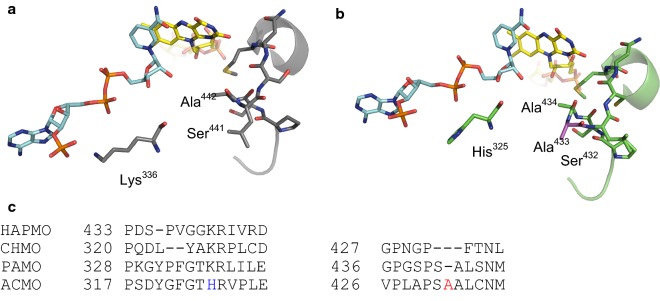
Comparison of the crystal structure of PAMO (**a**, PDB entry 2YLR) and a homology model of ACMO (**b**) depicting distinct residues neighboring the 2′-phosphate of NADP^+^ and the FAD and NADP^+^ are shown as stick models with the carbon atoms in yellow and cyan, respectively. **c** A sequence alignment of BVMOs showing variation in the active sites. Sequence variation in ACMO is highlighted in blue and red

To structurally rationalize the low NADP^+^ binding affinity we performed a sequence alignment of ACMO with related BVMOs and constructed a homology model of ACMO using MODELLER (version 9.20; Webb and Sali 2014). The model was created using a C65D variant of PAMO as a template (PDB entry 4d03), which shares 43% sequence identity with ACMO. The sequence alignment and ACMO model revealed sequence variation in the coenzyme binding cleft (Fig. 2). Typically, a lysine residue interacts with the 2′-phosphate of NADP(H) in BVMOs. This noncovalent interaction has been shown to improve the binding affinity for the coenzyme in addition to the enzyme’s preference for NADPH over NADH (Kamerbeek et al. 2004). In ACMO, a histidine residue (His^325^) replaces the lysine, a substitution that could potentially weaken the electrostatic interaction between the 2′ phosphate of the coenzyme and the protein. To test this hypothesis, His^325^ was substituted for a lysine. As shown in Tables 1 and 2, the H325K substitution lead to a significant improvement in coenzyme binding affinity as evidenced by the sixfold decrease in the *K*_m_ for NADPH and a 14-fold decrease in *K*_i_ for NADP^+^. The modeling exercise also revealed the insertion of an alanine (A433) in an active site bulge that has been shown to restrict the substrate scope of PAMO (Bocola et al. 2005; Reetz and Wu 2008). Deletion of this residue in ACMO led to a 130-fold decrease in the *k*_cat_/*K*_m_ for butanone, whilst the catalytic efficiency for cyclobutanone was unchanged. There was no increase in NADPH oxidation in the presence of acetone for this variant.

### NADP^+^ binding affinity for GSTACMO

**Fig. 3:**
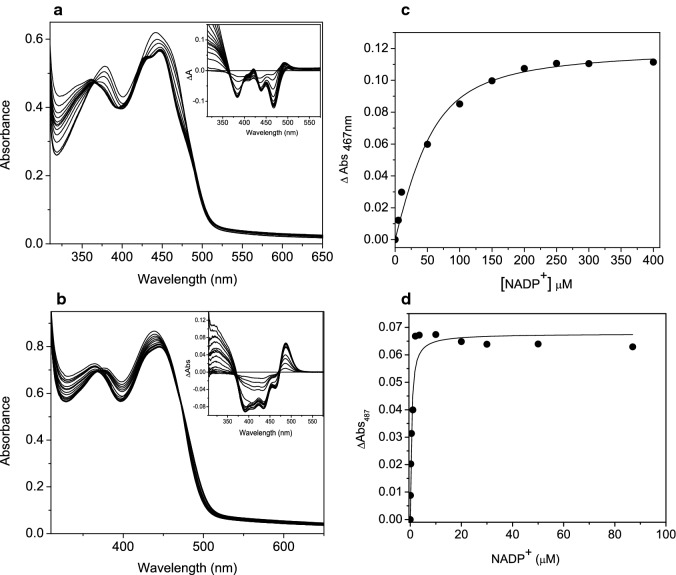
Flavin absorption spectra of GSTACMO (**a**) and H325K (**b**) were recorded in 50 mM HEPES–NaOH pH 7.5 upon titration with NADP^+^ (0–250 μM). The inset shows the difference spectra. Absorbance changes at 467 nm for wild type GSTACMO (**c**) and H325K (**d**) were plotted as a function of NADP^+^ concentration and the data were fitted to a quadratic binding isotherm (Eq. 1), which gave *K*_d_ values of 21.1 ± 4.1 μM (wild type) and 0.50 ± 0.04 μM (H325K)

The binding of NADP^+^ to PAMO and CHMO has been shown to induce notable shifts in the flavin absorbance spectra. This absorbance shift is attributed to displacement of a highly conserved catalytic arginine residue by the NADP^+^ nicotinamide ring as it docks over the xylene portion of the FAD isoalloxazine ring (Torres Pazmino et al. 2008; Sheng et al. 2001). In wild type CHMO and PAMO, binding of NADP^+^ causes the absorbance peak at 383 to shift to 366 nm and the peak at 440 nm to develop a more prominent absorbance shoulder at 480 nm. In GSTACMO, there is also a blue shift in the absorbance peak at 380 nm similar to that of related BVMOs (Fig. 3). However, in GSTACMO, the peak at 440 nm shifts to 450 nm with a sizable shoulder at 430 nm. The distinct spectral shifts observed in GSTACMO are likely to due to minor structural variations in the active site induced by the binding of the oxidized coenzyme. To determine the binding affinity of the coenzyme, the absorbance change at 467 nm was plotted against the concentration of NADP^+^ and the data were fitted to the equation describing the quadratic binding isotherm. For wild type GSTACMO, the dissociation constant for the GSTACMO-NADP^+^ complex was 21.1 ± 4.1 μM where as for the H325K variant it was 0.50 ± 0.04 μM.

### Reduction potential of the ACMO flavin cofactor

**Fig. 4:**
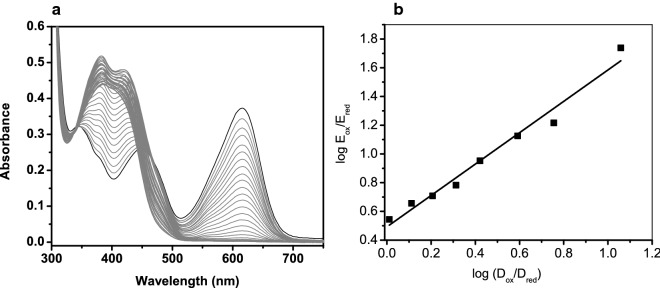
Reduction potential measurement of GSTACMO. **a** The combined absorbance spectra of GSTACMO and indigo disulfonate as both species are slowly reduced with xanthine/xanthine oxidase. The standard reduction potential value of the enzyme (E°), calculated from the y-intercept of the plot of log (*E*_red_/*E*_ox_) versus log (*D*_red_/*D*_ox_) shown in **b**, is − 166 mV ± 1 mV

The reduction potential of the enzyme was determined by reducing GSTACMO in the presence of indigo disulfonate, which served as a reference dye. Figure 4a shows the combined spectra of the dye and GSTACMO following incubation with the xanithine/xanthine oxidase reducing system and the redox mediator benzyl viologen. The concentrations of the oxidized and reduced forms of the dye and GSTACMO were determined at 610 and 458 nm, respectively, as described under “Materials and Methods”. A plot of log (*E*_red_/*E*_ox_) versus log (*D*_red_/*D*_ox_) has a slope of 1.0, which indicates that an equal number of electrons (i.e. two) were transferred between GSTACMO and indigo disulfonate (Fig. 4b). The midpoint potential of GSTACMO calculated from the y-intercept of the graph is − 166 ± 1 mV. Thus, GSTACMO is thermodynamically poised to accept a hydride ion from NADPH, which has a midpoint potential of − 320 mV.

### pH-dependence and thermostability

**Fig. 5:**
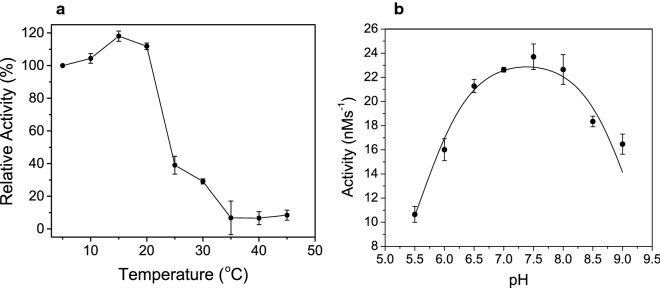
**a** Residual activity of GSTACMO following a 1 h pre-incubation of the enzyme at various temperatures. **b** pH-dependence of GSTACMO activity. Reactions were performed as described in “Materials and methods”

The thermostability of GSTACMO was measured by pre-incubating the enzyme at various temperatures (5–45 °C) for one hour and then measuring the residual activity at 25 °C (Fig. 5a). The enzyme elicited comparable residual activity at temperatures ≤ 20 °C. However, incubation of the enzyme at temperatures 25 °C and above resulted in > 60% loss of enzyme activity. These results demonstrate that GSTACMO is a relatively unstable protein. The pH-dependence profiles show a bell-shaped curve with maximal activity between pH 7 and 8 (Fig. 5b). The p*K*_a_ values of the ionizable groups responsible for optimal activity were determined by fitting the data to Eq. 5. The fitting routine revealed that enzyme activity is dependent on protonation of a single ionizable group with of p*K*_a_ of 5.6 ± 0.2 and ionization of group with a p*K*_a_ of 9.2 ± 0.3.

### Reductive-half reaction

**Fig. 6:**
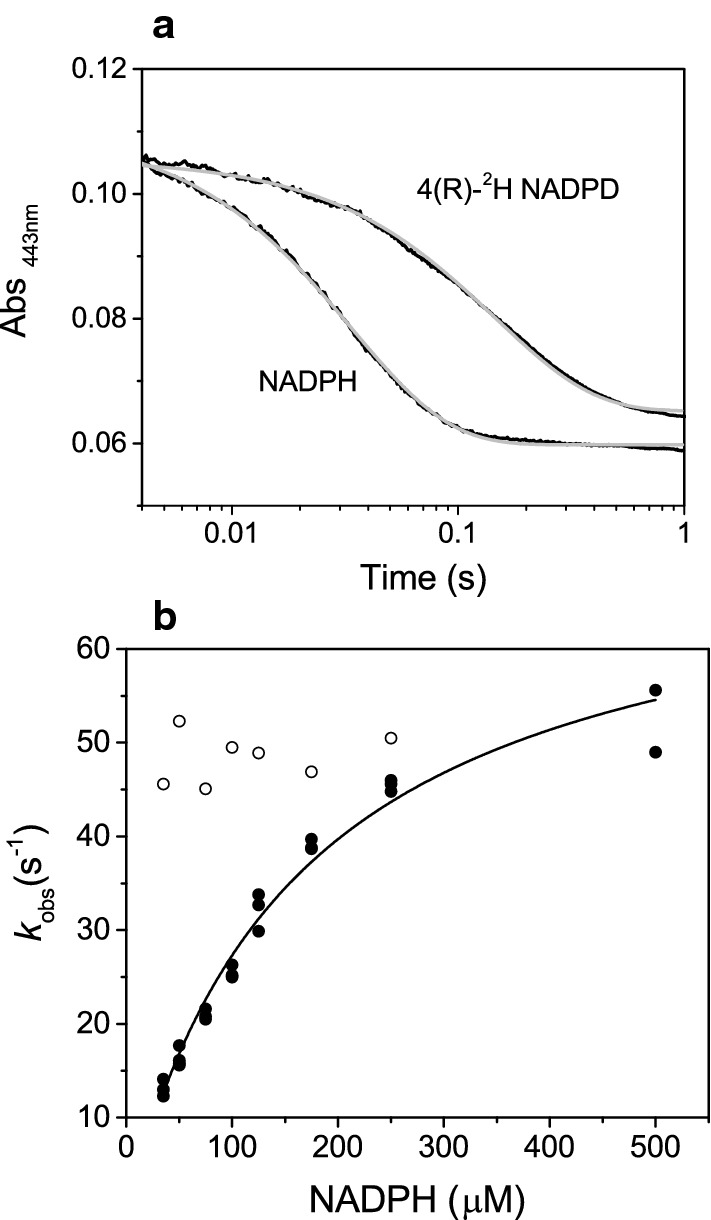
Single-wavelength stopped-flow experiments. **a** Stopped-flow single wavelength absorbance (443 nm) after mixing 20 μM of GSTACMO with 200 μM of NADPH or 4(*R*)-[4-^2^H]NADPH. Absorbance traces (black lines) were collected over 1 s (**a**) and fitted to standard single exponential equation (grey dotted line), which gave values of *k*_red1_ of 28.4 ± 0.5 s^−1^ (NADPH) and 7.5 ± 0.2 s^−1^ (4(*R*)-[4-^2^H]NADPH). **b** Dependence of *k*_red1_ on the concentration of NADPH of wild type ACMO (solid circles) and the H325K variant (open circles). The data were fitted to Eq. 6 and the results are shown in Table 2

The rate of NADPH-dependent reduction of GSTACMO was measured by following the absorbance decrease at 443 nm upon rapid mixing of the enzyme with a tenfold excess of coenzyme (Fig. 6a). The monophasic decay was fitted to a standard single exponential equation, which gave an observed rate constant (*k*_obs_) of 28.4 ± 0.5 s^−1^. To confirm that this initial kinetic phase involved transfer of a hydride ion from NADPH to the FAD cofactor, the reaction was repeated with 4(*R*)-[4-^2^H]NADPH. As shown in Fig. 6a, reduction of the enzyme with the deuterated coenzyme was significantly slower and a fit of the absorbance trace to a single exponential equation produced an observed rate constant of 7.5 ± 0.2 s^−1^ and a kinetic isotope effect of 3.8 ± 0.2.

Single-wavelength stopped-flow experiments were also used to determine if *k*_obs_ was affected by NADPH concentration. The stopped-flow absorbance changes were followed at 443 nm over 0.5 s and were fitted to a single exponential equation at concentrations of NADPH that were at least sevenfold greater than that of the enzyme concentration. The observed rate constant, *k*_obs_, exhibited a hyperbolic saturation dependence on NADPH concentration, and a fit of the data to Eq. 6 yielded a *K*_d_ of 121 ± 14 μM and a *k*_red_ (maximal rate constant of flavin reduction) of 59 ± 3 s^−1^ (Table 2; Fig. 6b). The H325K elicited a similar rate of NADPH-dependent flavin reduction as the wild type enzyme; however, the observed rate constant was not dependent on NADPH concentration, which is consistent with this variant having a higher affinity for the coenzyme.

### Oxidative-half reaction

**Fig. 7:**
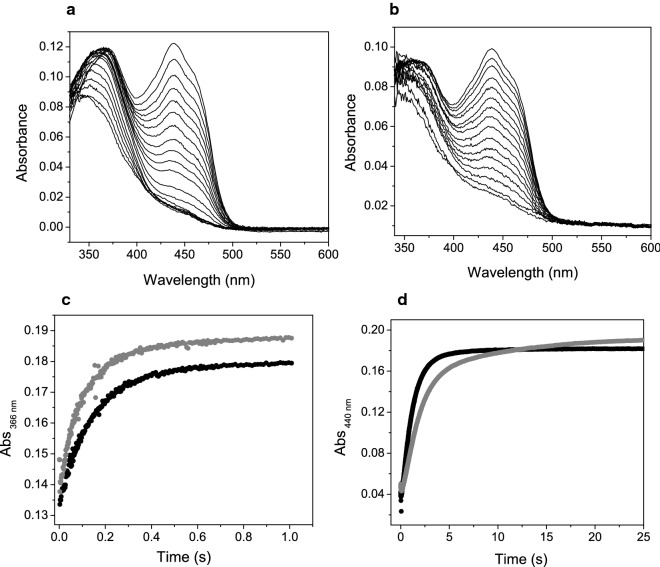
**a** Time-resolved absorbance spectra collected over 75 s following the re-oxidation of reduced GSTACMO (20 μM) in the presence 500 μM NADP^+^ with air-saturated buffer (50 mM HEPES pH 7.5, 20% glycerol). **b** The same experiment as performed as for panel A with the exception of 200 μM of butanone added to the air-saturated buffer. **c** Stopped-flow single wavelength traces at 366 nm following rapid mixing of reduced GSTACMO (20 μM) in the presence 500 μM NADP^+^ against air-saturated buffer (50 mM HEPES pH 7.5, 20% glycerol) with 200 μM of butanone (black circles) and without butanone (grey circles). Fitting the data to a single exponential equation gave observed rate constants of 6.5 ± 0.8 s^−1^ (with butanone) and 8.9 ± 0.2 s^−1^ (without butanone). **d** Stopped-flow single wavelength traces at 440 nm following rapid mixing of reduced GSTACMO (20 μM) in the presence 500 μM NADP^+^ against air-saturated 50 mM HEPES pH 7.5, 20% glycerol with 200 μM of butanone (black circles) and without butanone (grey circles). The absorbance traces with butanone were fitted to a single exponential equation giving an observed rate constant of 0.80 ± 0.01 s^−1^, and the absorbance traces without butanone were fitted to a double exponential giving rate constants of 0.47 ± 0.01 s^−1^ and 0.06 ± 0.01 s^−1^

**Fig. 8:**
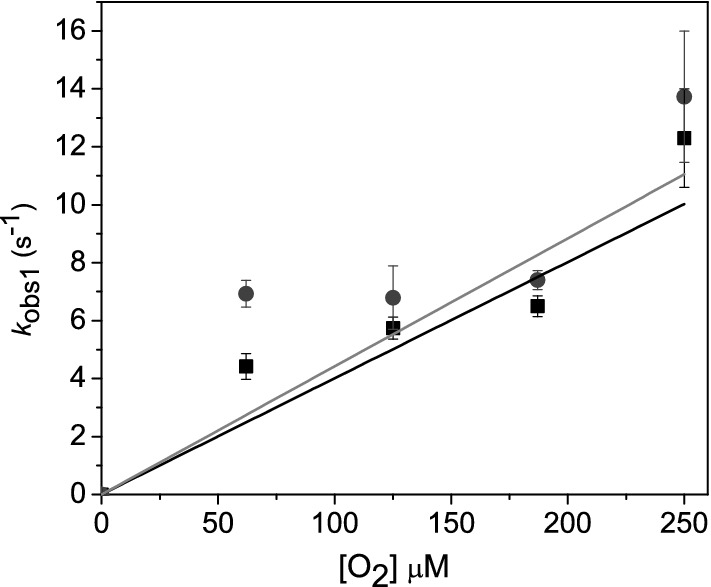
The dependence of the *k*_obs1_ measured at 366 nm on [O_2_]. The observed rate constants were plotted as a function of [O_2_] in the absence (closed squares) and presence of 200 μM butanone (closed circles) for GSTACMO. The slope of the lines were used to determine the bimolecular rate constants (*k*_oo-_ and *k*_oo-BT_) shown in Table 3

Stopped-flow spectroscopy was used to monitor the catalytic events of the oxidative half-reaction. In the first experiment, the reduced enzyme in the presence of 500 μM NADP^+^ was mixed with air-saturated buffer in the absence of ketone substrate. These mixing conditions lead to the formation of the C4a-peroxyflavin intermediate, which would subsequently decay to H_2_O_2_ and the fully oxidized flavin cofactor. For GSTACMO, the absorbance spectra were collected over 1.5 and 75 s on a log-time base and then subsequently combined; a selection of these spectra are shown in Fig. 7a. The time resolved spectra show initial formation of an absorbance peak at 366 nm, demarking formation of the C4a-peroxyflavin intermediate following O_2_ activation by the reduced FAD cofactor. The absorbance at 440 nm subsequently increases reflecting decomposition of the C4a-peroxyflavin intermediate and reformation of the oxidized FAD cofactor. If the reaction is repeated with the presence of 200 μM butanone in the air-saturated buffer, then formation of the C4a-peroxyflavin intermediate is less obvious, likely owing to its rapid decomposition in the presence of the carbonylic substrate (Fig. 7b). To extract rate constants for the kinetic events associated with the oxidative half reaction, we switched to single-wavelength mode with the stopped-flow apparatus, which enabled us to average multiple traces and acquire earlier data time points. In the absence of butanone, the absorbance changes at 366 nm were fit to a standard single exponential equation which gave an observed rate constant of 8.9 ± 0.2 s^−1^, while the observed rate constants at 440 nm were 0.47 ± 0.02 s^−1^ (*k*_ox1_) and 0.06 ± 0.01 s^−1^ (*k*_ox2_; Table 3). When the reaction was repeated in the presence of 200 μM butanone, then the observed rate constant at 366 nm was 6.5 ± 0.8 s^−1^ and at 440 nm, the single kinetic phase gave an observed rate constant 0.80 ± 0.01 s^−1^ (*k*_440_). The observed rate constant for the formation of C4a-peroxyflavin at 366 nm was shown to increase with oxygen concentration (Fig. 8). From a linear fit of the observed rate constant versus oxygen concentration, we obtained a second order rate constant of 49 mM^−1^ s^−1^ (*k*_oo-_) for the reaction between O_2_ and the reduced flavin. A similar bimolecular rate constant (*k*_oo-BT_) was obtained in the presence of butanone.

**Table 3:** Observed rate constants for the oxidative half reaction. The reactions were performed in 50 mM HEPES–NaOH, pH 7.5 at 25 °C

Without butanone			With butanone	
*k*_OO_^−^ (mM^−1^ s^−1^)^a^	*k*_ox1_ (s^−1^)^b^	*k*_ox2_ (s^−1^)^b^	*k*_OO_-_BT_ (mM^−1^ s^−1^)^a^	*k*_440_ (s^−1^)^c^
40.1 ± 5.1	0.47 ± 0.01	0.06 ± 0.01 s^−1^	44.2 ± 9.2	0.80 ± 0.01

^a^Determined from the slopes of a plot of the observe rate constants for the absorbance changes at 366 nm versus the concentration of dioxygen (Fig. 8)

^b^Determined from fitting a double exponential to the grey single wavelength absorbance trace at 440 nm shown in Fig. 7

^c^Determined from fitting a single exponential to the black single wavelength absorbance trace at 440 nm shown in Fig. 7

## Discussion

*Gordonia* sp. strain TY-5 is a bacterium that is capable of utilizing gaseous propane as the sole course of carbon during aerobic growth. ACMO is part of the propane degradation pathway present in the bacterium, for which acetone is a central intermediate. Although ACMO was initially identified as acting in the catabolism of acetone (Kotani et al. 2007), the enzyme elicits a 700-fold and 165-fold lower catalytic efficiency towards this substrate relative to butanone and cyclobutanone respectively. Although PAMO shares 43% sequence similarity with ACMO, PAMO does not consume acetone (de Gonzalo et al. 2005; Fraaije et al. 2005). Instead, PAMO preferentially oxidizes small aromatic ketones, such as phenylacetone. Mutagenesis studies of PAMO point to an active site loop comprising residues 440–446 as a structural feature that influences substrate specificity (Bocola et al. 2005; Reetz and Wu 2008). Deletion of residues S441 and A442 (PAMO numbering) resulted in an enzyme variant that was able to accept bulkier substrates (Bocola et al. 2005). Sequence alignment of PAMO and ACMO reveals that insertion of an alanine residue between S441 and A442, which may be a structural adaptation that restricts the ACMO active site and increase its catalytic efficiency towards smaller ketones such as acetone (Fig. 2). In ACMO, this residue (A433) improved catalytic efficiency towards small aliphatic ketones.

Stopped-flow spectroscopy was used to measure the rates of the reductive and oxidative half reactions. NADPH-dependent reduction of the ACMO FAD cofactor was relatively fast with an observed rate constant of ~ 60 s^−1^ at 25 °C. This rate constant is ~ 3–5-fold faster than that observed for CHMO and PAMO under similar experimental conditions (Ryerson et al. 1982; Torres Pazmino et al. 2008). However, the oxidative half reaction, particularly the formation of the C4a-peroxyflavin intermediate was considerably slower in ACMO. In CHMO and PAMO, the C4a-peroxyflavin adduct forms in < 10 ms at final O_2_ concentration of ~ 0.3 mM, and the second-order rate constant was determined to be 870 mM^−1^ s^−1^ for PAMO and 1700 mM^−1^ s^−1^ for CHMO. In contrast, the second order rate constant for the reaction between O_2_ and the reduced FAD in ACMO (49 mM^−1^ s^−1^) was 20–30-fold lower. The C4a-peroxyflavin is also not as stable in ACMO even in the presence of saturating amounts of NADP^+^ as it decayed with a rate constant of 0.47 s^−1^ in the absence of substrate. The instability of the C4a-peroxyflavin intermediate likely accounts for the relatively high rate of uncoupled NADPH oxidation (0.26 s^−1^) in ACMO. The NADPH oxidase activity of CHMO and PAMO is 50-fold and 150-fold lower, respectively, than that of the monooxygenase activity (Torres Pazmino et al. 2008; Sheng et al. 2001), but in ACMO it is only tenfold lower. It is unclear why this may be the case in ACMO, but it may be linked to the enzyme’s relatively weak binding affinity for NADP^+^.

Steady-state inhibition studies revealed that the NADP^+^ binding affinity is ~ 166 μM, similar to the dissociation constant for NADPH (120 μM) determined through stopped-flow experiments. By way of contrast, the *K*_d_ for NADPH is 0.7 μM and 11 μM for PAMO and CHMO, respectively (Torres Pazmino et al. 2008; Ryerson et al. 1982). Interestingly, the dissociation constant for the oxidized coenzyme determined through titration experiments was significantly lower at 21 μM. It unclear why a discrepancy is observed in the experimentally determined *K*_d_ values. It may have to do with the oxidation state of the coenzyme or enzyme. The stopped flow experiments measure the binding affinity of NADPH for the oxidized enzyme, steady-state inhibition studies measure the binding affinity of NADP^+^ for the reduced enzyme and the titration experiments measure the binding affinity of NADP^+^ to the oxidized enzyme. Previous studies have experimentally shown that the redox state of the enzyme can modulate the binding affinity for the pyridine nucleotide (van den Heuvel et al. 2005).

Crystal structures of CHMO and PAMO in complex with NADP^+^ show the coenzyme in an extended conformation, wedged between the Rossmann-fold of the NADP-domain and a loop within the FAD domain (Fig. 2). Residues coordinated to the coenzyme in PAMO are conserved in ACMO, with the exception of Lys^336^ (PAMO numbering), which coordinates to the 2′-phosphate of the coenzyme. Mutagenesis of the lysine in HAPMO (Lys^429^) demonstrates its importance in increasing the binding affinity of NADPH and ensuring that the enzyme preferentially selects for NADPH over NADH. In ACMO, the lysine to histidine substitution does not affect the enzyme’s preference for NADPH over NADH as both the wild type enzyme and the H325K variant were unable to catalyze the oxidation of NADH. The substitution does however strengthen the enzyme’s affinity for the 2′-phosphorylated coenzyme by sixfold.

In summary, we have shown that AMCO elicits the highest catalytic efficiency towards butanone and cyclobutanone. The enzyme also elicits a relatively weak binding affinity for NADP(H), which is partially attributed to sequence variation in the 2′-phosphate binding pocket. Reduced ACMO also reacts more slowly with O_2_ and is less efficient at stabilizing the C4a-peroxyflavin adduct compared to related BVMOs. As a consequence, ACMO elicits a relatively high NADPH oxidase activity compared to its monooxygenase activity. Finally, kinetic isotope studies support transfer of the pro*R* hydrogen. This implies that the *re*-face of the nicotinamide ring is planner with the FAD for reductive half reaction. Following hydride transfer the enzyme and cofactor undergo a conformational switch that places the *si*-face of the nicotinamide ring over the FAD, in position to stabilize reactive oxygenating intermediates of the oxidative half reaction.

## Additional file

**Additional file 1: Fig.S1.** A 10 % SDS-PAGE gel of recombinant ACMO. Lane 1, molecular weight markers; lane 2, crude extract; lane 3, glutathione-sepharose showing the GST-ACMO chimera and lane 4, recombinant ACMO following Q-sepharose chromatography.
