# Supplementary material for: Kinetic characterization of acetone monooxygenase from Gordonia sp. strain TY-5
Source: AMB Express. 2018 Nov 3;8:181. doi: 10.1186/s13568-018-0709-x (PMC6215540; doi:10.1186/s13568-018-0709-x)
Supplement: Supplementary file 1 — Additional file 1: Fig.S1. A 10 % SDS-PAGE gel of recombinant ACMO. Lane 1, molecular weight markers; lane 2, crude extract; lane 3, glutathione-sepharose showing the GST-ACMO chimera and lane 4, recombinant ACMO following Q-sepharose chromatography. [file 13568_2018_709_MOESM1_ESM.docx]

**Kinetic characterization of acetone monooxygenase from *Gordonia* sp. strain TY-5**

Osei Boakye Fordwour^†^, George Luka^‡^, Mina Hoorfar^‡^, and Kirsten R. Wolthers^†^*

^†^Department of Chemistry and ^‡^School of Engineering, University at the British Columbia, Okanagan Campus, 3247 University Way, Kelowna BC, V1V 1V7, CANADA

**Corresponding Author**:

*Email: kirsten.wolthers@ubc.ca; Phone: (250) 807 8663; Fax: (250) 807 9249

Keywords: Baeyer-Villiger monooxygenase, acetone monooxygenase, kinetic mechanism

stopped-flow spectroscopy, site-directed mutagenesis


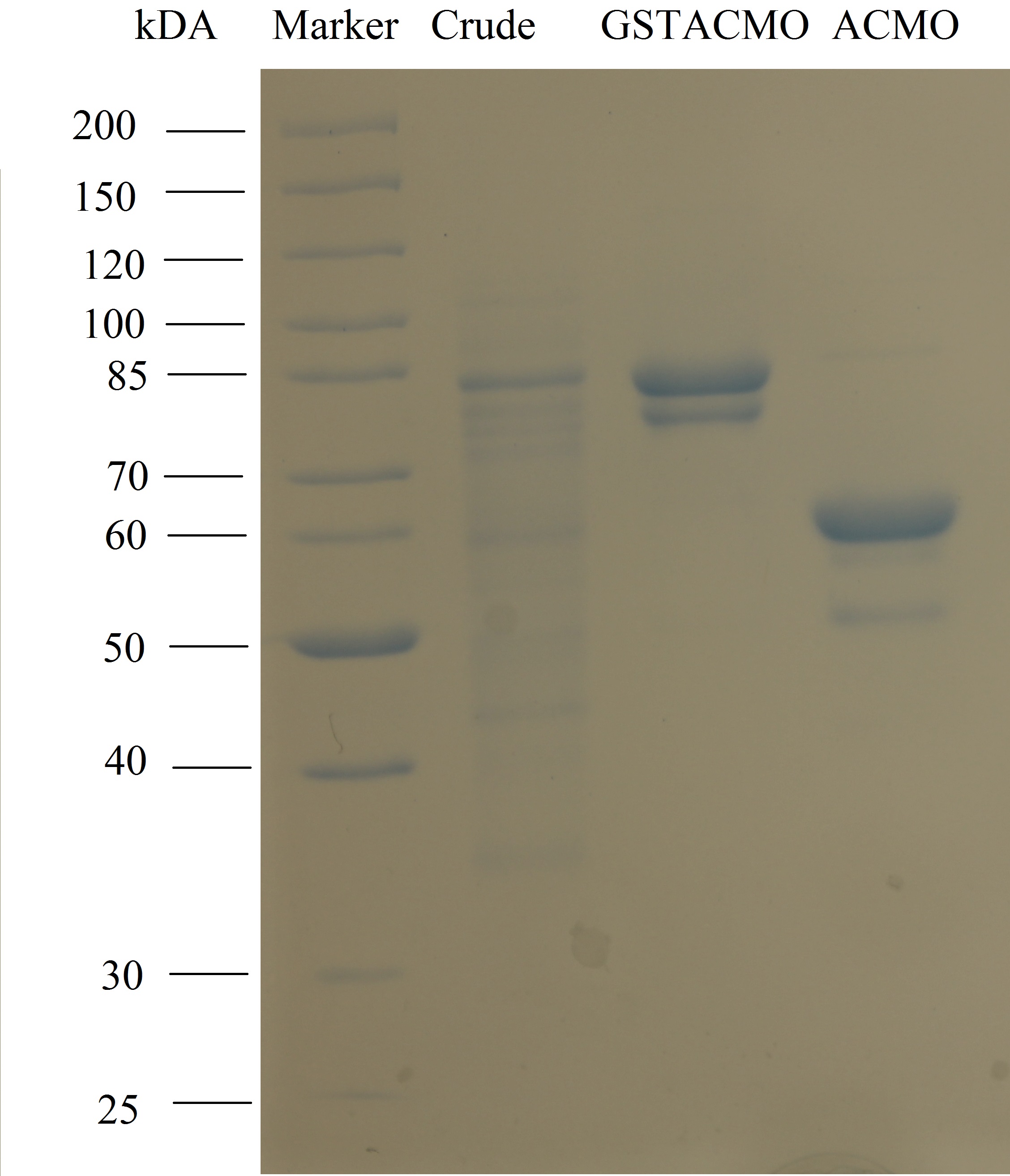
Fig S1. A 10 % SDS-PAGE gel of recombinant ACMO. Lane 1, molecular weight markers; lane 2, crude extract; lane 3, glutathione-sepharose showing the GST-ACMO chimera and lane 4, recombinant ACMO following Q-sepharose chromatography.

1 2 3 4
